# Efficient growth suppression in pancreatic cancer PDX model by fully human anti-mesothelin CAR-T cells

**DOI:** 10.1007/s13238-017-0472-9

**Published:** 2017-09-19

**Authors:** Hua Jiang, Bo Song, Peng Wang, Bizhi Shi, Qixiang Li, Mingliang Fan, Shengmeng Di, Jie Yang, Zonghai Li

**Affiliations:** 1 State Key Laboratory of Oncogenes and Related Genes, Shanghai Cancer Institute, Renji Hospital, Shanghai Jiaotong University School of Medicine, Shanghai, 200032 China; 2 CARsgen Therapeutics, Shanghai, 200231 China; 3 Crown Biosciences, Inc. Science & Technology Innovation Park, Taicang City, 215400 China; 4 Blood Engineering Laboratory, Shanghai Blood Center, Shanghai, 200051 China

**Dear Editor,**

Pancreatic cancer is a devastating disease ranked as the 4th leading cause of cancer-related deaths in the United States, and its incidence rate is increasing according to the latest statistics. The overall survival rates for patients with pancreatic cancer have not significantly improved over the past thirty years (Siegel et al., 2012; Simard et al., 2012). One of the reasons for the high mortality rates is the high resistance of pancreatic cancer to chemotherapy and radiation. Most patients are diagnosed at late stages of the disease. Approximately 15%–20% of patients diagnosed with pancreatic cancer are eligible for surgical resection, and 85% of these patients eventually experience relapse and ultimately cancer-related death (Siegel et al., 2012). In recent years, increasing evidence indicates that the fibro-inflammatory stroma is a source of cellular and molecular components contributing to tumor progression and metastasis (Feig et al., 2012; Waghray et al., 2013). Importantly, increased levels of stroma are positively related to a poor prognosis (Erkan et al., 2008). Despite the broader understanding of pancreatic cancer biology, gemcitabine, a chemotherapeutic approved for pancreatic cancer treatment approximately twenty years ago, still remains the standard of care (Burris et al., 1997). Thus, the development of novel treatment strategies for this devastating disease is urgently needed.

Immunotherapy based on T cells modified with a chimeric antigen receptor (CAR) has been demonstrated to be a promising strategy for cancer treatment. CAR T cells specifically recognize tumor-associated antigens and eliminate tumor cells in a non-major histocompatibility complex-restricted manner. Several pilot clinical trials using CAR T cells have recently been reported to have promising clinical outcomes, even in solid tumors (Brown et al., 2016; Kershaw et al., 2013). Mesothelin (MSLN) is a membrane protein that is overexpressed in many cancer types, including pancreatic cancers, and is expressed only at low levels on normal peritoneal, pleural, and pericardial mesothelial surfaces (Chang and Pastan, 1996). Previously, several types of MSLN-targeted CAR-T cells were developed and have been found to have impressive antitumor activities in mesothelioma and ovarian cancer models (Carpenito et al., 2009; Lanitis et al., 2012). However, there are no reports on the antitumor activities of anti-MSLNCAR-T cells toward pancreatic tumor xenograft models. No study has yet examined the use of CAR T cells in PDX models of pancreatic cancer. Therefore, it is necessary perform a preclinical evaluation of novel CAR T cells as a treatment for pancreatic cancer in PDX models.

In this study, we developed a novel fully human anti-mesothelin antibody. To investigate the binding properties of anti-MSLN antibody, we fist established the MSLN-overexpressed cell lines CHO-K1-MSLN and PANC-1-MSLN. The expression of mesothelin in these two established cell lines was confirmed by Western blotting (Fig. 1A). The fully human anti-MSLN antibody was screened from a fully human naïve antibody library by using phage display technology. The binding specificity of the anti-mesothelin antibody was tested on CHO-K1-MSLN and PANC-1-MSLN cells. The scFv proteins of anti-mesothelin antibody were produced transiently in FreeStyle™ 293F cells and purified by protein A affinity chromatography (Fig. S1). The results in Fig. 1C indicated that P1A6E and P3F2 scFv bound specifically to MSLN-expressing cells but not to cells without MSLN expression. Additionally, we compared the two fully human antibodies P1A6E and P3F2 with the SS1 and C10 antibodies. SS1 and C10 have a high binding affinity to mesothelin (Chowdhury and Pastan, 1999), and SS1 has also been found to be safe in patients when administered as a recombinant immunotoxin (Hassan et al., 2007). The results indicated that P1A6E and P3F2 had a significantly higher binding affinity than SS1 and C10 to MSLN-expressing cells (MFI value in PANC-1-MSLN cells: scFv-P1A6E: 327.5, scFv-P3F2: 308.8, scFv-SS1: 48.9 and scFv-C10: 46.8; MFI value in CHO-K1-MSLN cells: scFv-P1A6E: 452.3, scFv-P3F2: 445.1, scFv-SS1: 65.5 and scFv-C10: 80.2). The mean fluorescence intensity (MFI) of different scFv proteins bound cells as determined by flow cytometric analysis is shown in Fig. 1B. To test the affinity of antibody binding to mesothelin, we used Biacore Surface Plasmon resonance (SPR). The binding sensorgrams were collected at 25°C. The data were double-referenced by using reference flow cell 1 and the subtraction of a preceding buffer blank with BiaEvaluation (v4.1). The processed binding curves were fitted to the Langmuir model for a 1:1 binding stoichiometry. Representative Biacore data are shown in Fig. 1D. All binding data are summarized in Table S1.

**Figure 1:**
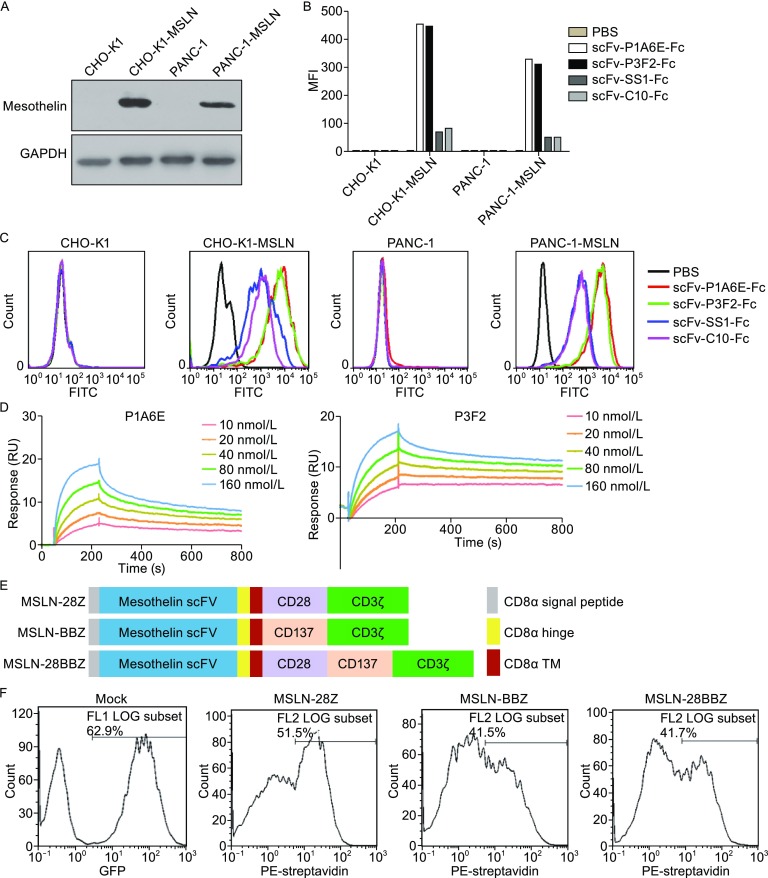
**Binding properties of anti-mesothelin antibody and CAR constructs on primary human T cells**. (A) Mesothelin expression in the established cell lines. Cell extracts from mesothelin-transfected cells were subjected to Western blot analysis. The blot was incubated with a mouse monoclonal antibody mesothelin (K1). GAPDH was used as a loading control. (B) The mean fluorescence intensity (MFI) of different scFv proteins bound cells was determined by flow cytometric analysis. (C) Binding specificity of the anti-mesothelin antibody to MSLN-transfected CHO-K1 or PANC-1 cells. (D) Affinity measurements of antibody binding to mesothelin through Biacore Surface Plasmon resonance. (E) A schematic diagram showing the MSLN-specific CAR used in this study. (F) The primary human T cells efficiently express MSLN-specific CAR, as measured by flow cytometry. Mock T cells were demonstrated by assessing the expression of eGFP

Then, we generated the CAR-MSLN T cells by lentiviral vector transduction. Three types of CARs that incorporated a human MSLN-specific scFv and CD28/CD3ζ, CD137/CD3ζ or CD28/CD137/CD3ζ signaling domains (MSLN-28Z, MSLN-BBZ, MSLN-28BBZ) were constructed. These recombinant lentiviral constructs are schematically shown in Fig. 1E. To determine the expression of MSLN-CAR on the surface of genetically modified T cells, the lentiviral vectors encoding the MSLN-targeted CARs, including MSLN-28Z, MSLN-BBZ and MSLN-28BBZ, were efficiently transduced into human primary T cells. The same lentivirus encoding eGFP was also used to transduce T-cells (referred to as mock T cells). On day 7 after transduction, the expression of different CARs in the transduced T cells was determined by flow cytometry by using biotinylated anti-human-F(ab′)2 fragment antibody and PE-conjugated streptavidin. The CAR transduction efficiencies ranged from 41.5%–51.5% (Fig. 1F). The transduced efficiency of mock T-cells was demonstrated by eGFP expression (Fig. 1F).

To determine whether MSLN-targeted CAR T cells specifically recognize and kill MSLN-positive pancreatic cancer cells, cytotoxicity assays were performed by incubating the transduced T-cells with PANC-1 cells with or without MSLN expression. As shown in Figure 2A, MSLN-28Z, MSLN-BBZ, and MSLN-28BBZ T-cells efficiently killed MSLN-positive pancreatic cancer cells, but almost no lysis was observed in PANC-1 cells not expressing MSLN. Control mock T-cells did not lyse the target cells tested. Notably, MSLN-28Z and MSLN-28BBZ had higher tumor lysis ability than MSLN-BBZ T-cells at an effector:target ratio of 3:1 (*P* < 0.05) (Fig. 2A). Therefore, in subsequent experiments, we used MSLN-28Z and MSLN-28BBZ T-cells. To explore whether cytokines are produced by different formats of the MSLN-specific CAR-T cells to respond to pancreatic cancer cells, the major cytokines (TNF-α, IL-2, and IFN-γ) correlated with the function of the CAR-T cells were examined. mock T-cells were used as a negative control. As shown in Figure 2B, in the presence of MSLN-negative PANC-1 cells, very low levels of the indicated cytokines were observed. By contrast, increased amounts of all three cytokines were detected in MSLN-specific CAR-T cells incubated with the MSLN-expressing PANC-1 cells. The level of IFN-γ was similar among the three CARs. However, higher levels of IL-2 and TNF-α were secreted by the MSLN-28Z and MSLN-28BBZ T-cells than the MSLN-BBZ T-cells under co-culture with MSLN-positive pancreatic cancer cells (*P* < 0.001, Fig. 2B).

**Figure 2:**
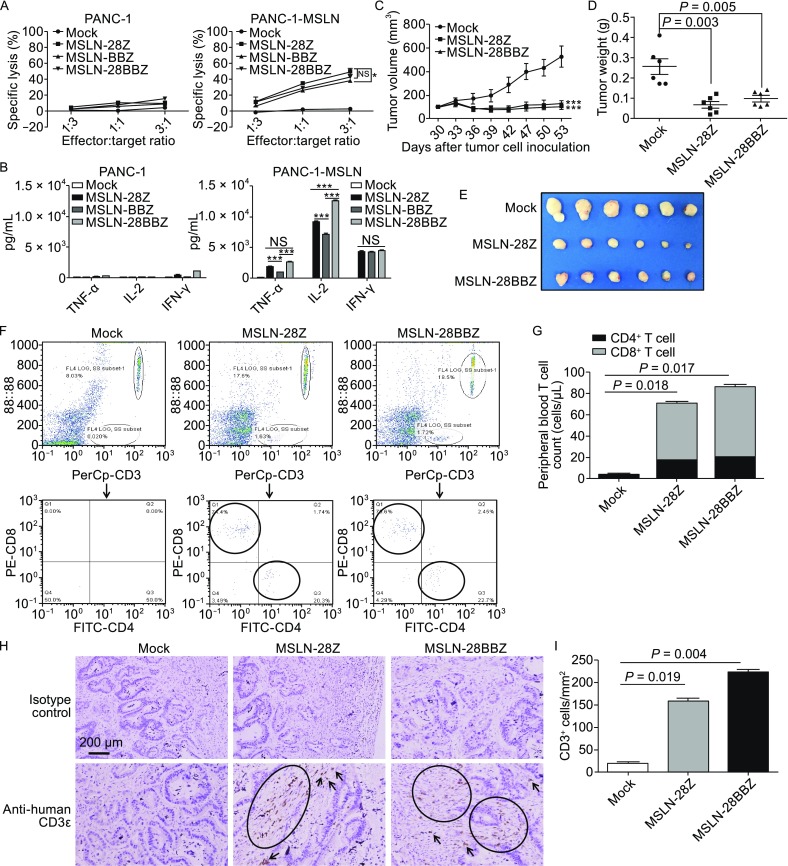
***In vitro*** **cytotoxic activity and cytokine release of MSLN-targeted CAR T cells and antitumor activity of the modified T cells against established pancreatic cancer PDX tumor models** ***in vivo***. (A) Modified T cells were coincubated with tumor cells for 18 h at different effector: target ratios. Cell lysis was determined through a standard nonradioactive cytotoxic assay. Each data point is the mean ± SEM of triplicates. (B) The modified T cells were cocultured with tumor cells for 24 h. The levels of TNF-α, IL-2, and IFN-γ in the supernatants were evaluated by ELISA. The results are representative of triplicates. Statistically significant difference is marked by asterisks (*, *P* < 0.05; ***, *P* < 0.001). (C) NOD/SCID mice were inoculated subcutaneously with pancreatic cancer PDX tumors on day 0. On day 30, when the tumor volumes reached 100 mm^3^, T cells were injected i.v. with a single dose of 1 × 10^7^ MSLN-28Z and MSLN-28BBZ CAR-T cells or mock transduced T cells. Data are presented as mean tumor volume ± SEM. Statistically significant differences of MSLN-28Z vs. mock T-cells or MSLN-28BBZ vs. mock T-cells are marked by asterisks (***, *P* < 0.001). (D) On day 53 after pancreatic cancer PDX tumor inoculation, mice were euthanized. The tumor weight was measured. Efficacy was evaluated by measuring the reduction of the mean tumor weight relative to mock control (*P* < 0.01). (E) The images of xenograft tumors treated with different CAR-T cells. (F) The persistence of CAR-T cells *in vivo*. The flow cytometric analysis of human CD4^+^ and CD8^+^ T cells from mice bearing pancreatic cancer PDX tumors treated with the indicated genetically modified T cells. (G) The quantities of circulating human CD4^+^ and CD8^+^ T cells. The mean cell concentration (cells/μL ± SEM) for mice in the untransduced or modified T cell treatment groups and *P* values are shown. (H) MSLN-CAR T cells infiltrate into pancreatic cancer PDX tumors. Tumors were collected from mice bearing pancreatic cancer PDX subcutaneous tumors treated with MSLN-28Z, MSLN-28BBZ CAR-T cells and mock T cells. Formalin-fixed, paraffin-embedded tumor sections were consecutively cut and stained for human CD3 expression (brown). The images were obtained under 200× magnification. The scale bar is 200 μm. (I) The quantities of infiltrated CAR-T cells in pancreatic cancer PDX subcutaneous tumors. Data are expressed as mean ± SEM

Because the MSLN-28Z and MSLN-28BBZ CAR-T cells displayed higher tumor lysis capacity and greater cytokine secretion than MSLN-BBZ CAR-T cells* in vitro*, they were selected for* in vivo* antitumor assays. NOD/SCID mice bearing pancreatic cancer PDX xenografts were used. The expression of MSLN on these PDX tumors was confirmed by immunostaining (Fig. S2). On day 30, when the tumor volumes reached 100 mm^3^, T cells were injected i.v. with a single dose of 1 × 10^7^ MSLN-28Z and MSLN-28BBZ CAR-T cells or mock transduced T cells. As shown in Figure 2C, compared with the control groups, the MSLN-28Z and MSLN-28BBZ CAR-T cells significantly suppressed the growth of pancreatic cancer PDX engrafted tumors (*P* < 0.001). On day 53 after pancreatic cancer PDX tumor inoculation, efficacy was evaluated by measuring the decrease in the mean tumor weight relative to the mock control. Mice treated with MSLN-28Z and MSLN-28BBZ T cells had a lower tumor weight than those in the mock control group (MSLN-CD28Z vs. mock, *P* = 0.003; MSLN-CD28BBZ vs. mock, *P* = 0.005; Fig. 2D). These results suggested that MSLN-28Z and MSLN-28BBZ CAR-T-cells can efficiently eliminate pancreatic cancer PDX tumor xenografts* in vivo*.

Previous studies have demonstrated that the persistence of transferred T cells * in vivo* is highly correlated with tumor suppression (Chapuis et al., 2012). To investigate the persistence of CAR T-cells* in vivo*, pancreatic cancer PDX tumor-bearing mice were euthanized to test the persistence and infiltration of T-cells in the mouse peripheral blood and tumor tissue 8 days after T cell infusion. As show in Figure 2F, a significant increase in both CD4^+^ and CD8^+^ T-cells was found in the MSLN-28Z and MSLN-28BBZ groups compared with the mock group, and the T cell numbers were highest in the group treated with MSLN-28BBZ CAR T cells (MSLN-28Z vs. mock, *P* = 0.017; MSLN-28BBZ vs. mock, *P* = 0.018; Fig. 2G). In addition, the infiltration of human T-cells was further certified by CD3^+^ T-cell immunostaining of pancreatic cancer PDX tumors treated with MSLN-specific CAR-T cells. The results showed that human CD3^+^ cells accumulated in residual tumors 8 days after i.v. T cell administration, whereas fewer T-cells were detected in tumors treated with mock transduced T-cells (MSLN-28Z vs. mock, *P* = 0.019; MSLN-28BBZ vs. mock, *P* = 0.004; Fig. 2H and 2I). These data demonstrated that the transferred MSLN-specific CAR-T cells survived* in vivo* and trafficked into mesothelin positive pancreatic cancer PDX tumors.

It has been reported that MSLN-CAR T cells using the mouse anti-MSLN antibody SS1 can cause anaphylaxis in humans (Maus et al., 2013); therefore, it is necessary to develop human or humanized anti-MSLN CAR-T cells to reduce the immunogenicity of CAR-T cells. In this study, we developed fully human anti-MSTL CAR-T cells. Furthermore, to mimic the hostile stroma microenvironment, a pancreatic cancer patient derived xenograft (PDX) model expressing mesothelin was investigated *in vivo* for antitumor activities of anti-MSTL CAR-T cells. Unlike cancer cell lines, primary tumor cells in PDX models are directly derived from human tissues and are not subjected to frequent high-serum environments and passages. Therefore, PDX models are more biologically stable during passaging in mice in terms of mutational status, gene expression patterns, and tumor heterogeneity (Jin et al., 2010).

Together, our results demonstrated that the anti-mesothelin CAR-T cells efficiently inhibit the growth of pancreatic cancer PDX and may be a potential novel treatment strategy for patients with pancreatic cancer.

## **FOOTNOTES**

The study was funded by the Supporting Programs of Shanghai Subject Chief Scientist (No. 16XD1402600), Shanghai Science and Technology Innovation Action Plan (No. 16DZ1910700), the National Natural Science Foundation of China (Grant No. 81502672), and the Grant-in-Aid for Young Scientists Foundation of Shanghai Cancer Institute (No. sb201601 and sb201603)

Dr. Zonghai Li has ownership interests of anti-mesothelin CAR-T and owns stock in CARsgen Therapeutics Co. Ltd. Hua Jiang, Bo Song, Peng Wang, Bizhi Shi, Qixiang Li, Mingliang Fan, Shengmeng Di, and Jie Yang declare that they have no conflict of interest.

For studies with animals, mice were housed and treated according to protocols approved by the Shanghai Medical Experimental Animal Care Commission. All institutional and national guidelines for the care and use of laboratory animals were followed.

## Electronic supplementary material

Below is the link to the electronic supplementary material.

Supplementary material 1 (PDF 1052 kb)
