# Supplementary material for: Efficient growth suppression in pancreatic cancer PDX model by fully human anti-mesothelin CAR-T cells
Source: Protein Cell. 2017 Sep 19;8(12):926–31. doi: 10.1007/s13238-017-0472-9 (PMC5712292; doi:10.1007/s13238-017-0472-9)
Supplement: Supplementary file 1 — Supplementary material 1 (PDF 1052 kb) [file 13238_2017_472_MOESM1_ESM.pdf]

**Supplementary Information**

Materials and methods

Tables S1

Figures S1-3

## Materials and methods

## Cell lines

The human pancreas ductal adenocarcinoma cell line PANC-1 and human embryonic kidney 293T cell lines were obtained from the American Type Culture Collection (ATCC, Manassas, VA, USA). The Chinese hamster ovary cell line CHO-K1 was obtained from the Type Culture Collection of the Chinese Academy of Sciences (Shanghai, China). PNAC-1-MSLN and CHO-K1-MSLN cells were established by lentiviral transduction with the pCMV-mesothelin gene, constructed by our laboratory, into PNAC-1 and CHO-K1 cell lines. These cells were cultured in Dulbecco's modified Eagle's medium (DMEM) supplemented with 10% fetal bovine serum (FBS) (Life Technologies Corporation, Carlsbad, CA) and 100 μg/ml penicillin, and 100 U/ml streptomycin (Invitrogen, Carlsbad, CA). All cells were maintained at 37℃ in humidified air with 5% $CO_2$. FreeStyle™ 293F cells were obtained from Thermo Fisher Scientific and were incubated at 37℃ with 8% $CO_2$.

## Selection of anti-mesothelin antibodies from the phage library

The fully human anti-MSLN antibody was screened from a fully human naïve antibody library by using phage display technology. The recombinant mesothelin was biotinylated with EZ-LinkTM sulfo-NHS-Biotin (Pierce, Rockford, IL) according to the manufacturer's instructions. The biotinylated antigen (final concentration $10^{-7}$ M) was incubated with $10^{12}$ phage particles (a naïve scFv library) in 1 ml 1% BSA for 40 min on a shaker at room temperature. The phage-antigen complex was captured in avidin-coated or streptavidin-coated Maxisorp wells (Thermo Fisher Scientific, Waltham, MA). The phage-antigen mix was distributed in eight wells and incubated for 20 min on a shaker at RT. The wells were rinsed 10 times with phosphate-buffered saline (PBS) 0.1% Tween-20 and 10 times with PBS. Bound phage was eluted by incubation with 200μl/well 100mM triethylamine for 5-10 min. The eluted phage was used to infect exponentially growing Escherichia coli TG-1.

## Expression and purification of P1A6E and P3F2 scFv-Fc fusion protein

To express P1A6E and P3F2 scFv-Fc fusion proteins in FreeStyle™ 293F cells, two expression vectors were constructed separately. Primers V5-P1A6E-F (5'-ACAGTGCTAGCACAGGTACAGCTGGAACAG-3') and V5-P1A6E-R (5-'TTGTCGGATCCACCTAGGACGGTGACC-3') were used to amplify the scFv fragment of P1A6E from a phagemid, and primers V5-P3F2-F (5'-ACAGTGCTAGCACAGATGCAGCTAGTGC-3') and V5-P3F2-F (5'-TTGTCGGATCCACGTTTGATCTCCAGC-3') were used to amplify the scFv fragment from a phagemid. The scFv fragment was digested with NheI and BamHI and cloned into the expression vector pCMV-V5-Fc, which contains the Fc

(hinge+CH2+CH3) part of IgG1 (purchased from Shanghai raygene biotechnology). FreeStyle™ 293F cells were transfected with the expression vector according to the manufacturer's instructions. The cells were incubated at 37℃ with 8% $CO_2$ for 6-7 days. ScFv-Fc fusion proteins were collected and the cell culture supernatants were purified by protein A affinity chromatography. The control antibodies SS1 and C10 (US7081518B1) were expressed and purified through the same method.

**Surface plasmon resonance**

Kinetic measurements were performed by using surface plasmon resonance with a Biacore T200™. The 1×HBS-EP+ buffer was chosen as the running buffer. Approximately 1000 response units (RU) anti-human Fc antibody (GE, #BR100839) were immobilized on a CM5 sensor chip through standard amine coupling. Purified scFv-Fc was injected at predetermined concentrations to achieve 100 RU for the captured scFv-Fc. Association and dissociation rate constants were determined by injection of a concentration range of the recombinant mesothelin at a constant flow rate (10μl/min). The channel was regenerated by injection of 3 M $MgCl_2$ over 2 min.

**Western blot analysis**

To confirm the levels of mesothelin expression in cell lines of stablely transfected human mesothelin protein, $3 \times 10^6$ cells were lysed in 200μl lysis buffer for 60 min on ice. Cell lysate was then removed by centrifugation at 12,000 × g for 10 min. Each sample was denatured under reducing conditions and electrophoresed by 12% SDS–PAGE. The samples were then transferred to a nitrocellulose membrane (Bio-Rad, Hercules, CA) and immunoblotted with a mouse monoclonal antibody mesothelin (K1) (Santa Cruz Biotechnology, Santa Cruz, CA). GAPDH was used as a loading control. The blots were incubated with horseradish peroxidase-conjugated anti-mouse IgG (Kangchen Biotech, Shanghai, China) and detected using the ECL western blot analysis system (Pierce, Thermo Scientific, Rockford, IL) in accordance with the manufacturer's instructions.

**Mesothelin-specific CARs construction**

The sequence encoding the MSLN-scFv antibody in the VL-VH orientation was obtained by polymerase chain reaction (PCR) amplification from a plasmid encoding the scFv-P3F2-Fc. As shown in Fig. 2A, the MSLN-28Z CAR contained the human CD8α signal peptide, followed by the MSLN-scFv linked in-frame to the hinge domain of the CD8a molecule, the transmembrane region of the human CD28 molecule, and the intracellular signaling domains of the CD28 and CD3ζ molecules. The MSLN-BBZ CAR contained the human CD8α signal peptide, followed by the MSLN-scFv linked in-frame to the hinge domain of the CD8a molecule, the transmembrane region of the human CD8 molecule, and the intracellular signaling domains of the CD137 and CD3ζ molecules. The MSLN-28BBZ CAR contained the human CD8α signal peptide, followed by the MSLN-scFv linked in-frame to the hinge domain of the CD8a

molecule, the transmembrane region of the human CD28 molecule, and intracellular signaling domains comprising CD28, CD137, and CD3ζ. The nucleotide sequences of the human CD8α hinge and transmembrane region of the human CD28 molecule and intracellular signaling domains of CD28, 4-1BB, and CD3ζ were assembled in-frame to produce three different configurations referred to as 28Z, BBZ or 28BBZ. The 28Z, BBZ or 28BBZ fragments were produced by PCR amplification using a plasmid encoding the corresponding fragments (Gao et al., 2014) as a template. The scFv DNA fragments and 28Z, BBZ or 28BBZ fragments were then recombined using PCR. These fragments were designed to have a MluI site at the 5' end and a SalI site at the 3' end. The synthesized fragments were digested with MluI and SalI restriction enzymes (New England Biolabs, USA) and ligated individually into the similarly digested pRRLSIN.cPPT-GFP.WPRE vector plasmid. The sequence integrity of all vectors described in this paper was confirmed by DNA sequencing. The Mock construct was transduced using a pRRLSIN.cPPT-GFP.WPRE lentiviral vector.

**Lentivirus production**

The 293T cells were seeded at $6 \times 10^6$ per 10-cm dish before transduction. The next day, 293T-cells were transfected with pRRLSIN.cPPT-GFP.WPRE vector (mock) or the different recombinant expression vectors in addition to the lentiviral packaging plasmid pMDLg/pRRE, pRSV-Rev and envelope-expressing plasmid pCMV-VSV-G (from Addgene) by using a polyethylenimine-based DNA transfection reagent. The viral supernatants were harvested at 48 or 72 h after transfection. Then, the lentiviral particles were concentrated 30-fold by ultracentrifugation (Beckman Optima™ XL-100 K, Beckman, Germany) for 2 h at 28,000 rpm.

**Transduction and culture of primary T cells**

Peripheral blood mononuclear cells (PBMCs) derived from human donors were provided by the Shanghai Blood Center. PBMC cells were cultured in AIM-V medium (Invitrogen, Carlsbad, CA) with 2% human AB serum (Huayueyang Biotechnology, China) and recombinant human IL2 (Huaxin High Biotech, China). For the transduction of primary T cells, PBMCs were stimulated for 48 h with anti-CD3/anti-CD28 antibodies immobilized on tosyl-activated paramagnetic beads (Invitrogen, Carlsbad, CA) before infection. After stimulation, the T cells were transduced with lentivirus particles in 24-well plates coated with RetroNectin (TaKaRa, Japan). The transduced T cells were cultured at a concentration of $5 \times 10^5$ cells/ml in the presence of rhIL2 (300 IU/ml).

**Flow cytometric analysis**

To detect scFv binding to target cells, $2 \times 10^5$ cells were collected by centrifugation and stained with anti-mesothelin scFv-Fc antibodies (10μg/ml) for 1 h at 4℃ followed by FITC conjugated goat anti-human secondary antibody (Kang-Chen Bio-tech, Shanghai, China) in the dark for 45 min at 4℃.

To measure CAR expression, the various genetically modified T cells were detected

by using a biotinylated anti-human-F(ab')2 fragment (1:50, Jackson) and incubated at 4℃ for 45 min. After being washed with FACS buffer, cells were then incubated with PE-conjugated streptavidin for 45 min at 4℃ (eBioscience, San Diego, CA). For analysis of the absolute number of human T cells, $CD4^+$ and $CD8^+$ T cells were quantified using TruCount tubes (BD Biosciences, San Jose, CA) as described in the manufacturer's instructions. Fluorescence was assessed using a BD FACSCelesta Flow cytometer and data were analyzed with FlowJo7.6 software.

**Cytotoxicity assays in vitro**

Two human pancreas cells were co-cultured with the genetically modified T cells at different effector: target ratios of 3:1, 1:1 and 1:3. After 18 h of culture, the level of released LDH in the supernatant was measured using a CytoTox 96® non-radioactive cytotoxicity Kit (Promega, Madison,WI) as previously described (Gao et al., 2014). This experiment was repeated three times with consistent results.

**Cytokine release assay**

Cytokine measurements were performed by co-culturing transduced T cells with cancer cells in 96-well culture plates. After co-culture for 24 h, the IFN-γ, TNF-α and IL-2 cytokines secreted by the genetically modified T cells stimulated by the target cells were measured using an ELISA kit according to the manufacturer's instructions (MultiSciences Biotechnology, Hangzhou, China).

**Pancreatic cancer PDX tumor models**

The immunocompromised mice engrafted with pancreatic cancer patient-derived xenograft were kindly provided from Crown Bioscience. The 6-8-week-old female, non-obese diabetic/severe combined immunodeficient (NOD/SCID) mice were housed and treated under specific-pathogen-free conditions and used for tumor engraftment. Briefly, the tumors were sliced into 3 mm × 3 mm × 3 mm fragments and inoculated subcutaneously on the right flank. When the tumor burdens were approximately 100 $mm^3$, the mice were randomly separated into three groups (n = 6) and injected intravenously (i.v.) with different CAR-T cells ($1\times10^7$ CAR-T cells/mouse) after lymphocyte depletion with cyclophosphamide (100mg/kg). The tumor dimensions were measured twice weekly with calipers, and the tumor volumes were calculated using the formula $V = \pi/6 \times (\text{length} \times \text{width}^2)$, where the length is the greatest longitudinal diameter and the width is the greatest transverse diameter (Goldstein et al., 1995). Mice were euthanized when their body weight loss was greater than 20% of the initial weight, when they exhibited inability to ambulate, or when tumor ulceration was observed in the control groups. NOD/SCID mice were housed and treated according to protocols approved by the Shanghai Medical Experimental Animal Care Commission.

**Immunohistochemistry**

To assess the infiltration of human T cells into xenograft tumors, formalin-fixed, paraffin-embedded tumor tissues were immunostained using an anti-CD3 antibody (Thermo Fisher Scientific, Waltham, MA). A normal rabbit IgG served as an isotype control. The procedures were performed as previously described (Gao et al., 2014).

Briefly, after deparaffinization and rehydration, the sections were exposed to 3% $H_2O_2$ in methanol to eliminate endogenous peroxidase activity. Bovine serum albumin (1%) was used to block the sections for 30 min at room temperature (RT). The primary rabbit anti-human CD3 monoclonal antibody was incubated overnight at 4℃. The sections were then washed with PBS and incubated with an HRP-conjugated goat anti-rabbit secondary antibody (Kangchen Biotech, Shanghai, China) for 45 min at RT. The sections were visualized using a diaminobenzidine staining kit (Tiangen Biotech, Beijing, China) and then counterstained with hematoxylin, dehydrated, cleared, mounted and photographed. DAB-immunostained sections were analyzed by bright-field microscopy using an Olympus microscope (OLYMPUS IX71, Japan). $CD3^+$ cells were quantified by measuring the number of stained T-cells in each section from three mice in each group by using Image-Pro Plus software. The mean count of the three areas was taken and expressed as the absolute number of CD3+ cells per 0.95 $mm^2$ (×200 field).

**Statistical analysis**

All data are presented as the mean ± standard error of the mean (SEM). Data were analyzed using a two-way ANOVA with a Bonferroni post-test for multi-sample comparisons for multi-sample comparisons or Student's t test for two sample comparisons. GraphPad Prism 5.0 was used for the statistical calculations. A *P* value less than 0.05 was considered significant.

Gao, H., Li, K., Tu, H., Pan, X., Jiang, H., Shi, B., Kong, J., Wang, H., Yang, S., Gu, J., and Li, Z. (2014) Development of T cells redirected to glypican-3 for the treatment of hepatocellular carcinoma. Clinical cancer research : an official journal of the American Association for Cancer Research 20: 6418-6428.

Goldstein, N. I., Prewett, M., Zuklys, K., Rockwell, P., and Mendelsohn, J. (1995) Biological efficacy of a chimeric antibody to the epidermal growth factor receptor in a human tumor xenograft model. Clinical cancer research : an official journal of the American Association for Cancer Research 1: 1311-1318.

**Table S1. Calculated KD values of these two antibodies or scFvs for affinity of binding to mesothelin**

| Samples | ka (1/Ms) | kd (1/s) | KD (M) |
|---|---|---|---|
| P1A6E(IgG) | 2.88E+05 | 1.16E-03 | 4.04E-09 |
| P3F2(IgG) | 1.10E+05 | 7.89E-04 | 7.17E-09 |
| P1A6E(scFv-Fc) | 8.25E+04 | 1.56E-03 | 1.89E-08 |
| P3F2(scFv-Fc) | 5.77E+04 | 9.35E-04 | 1.62E-08 |

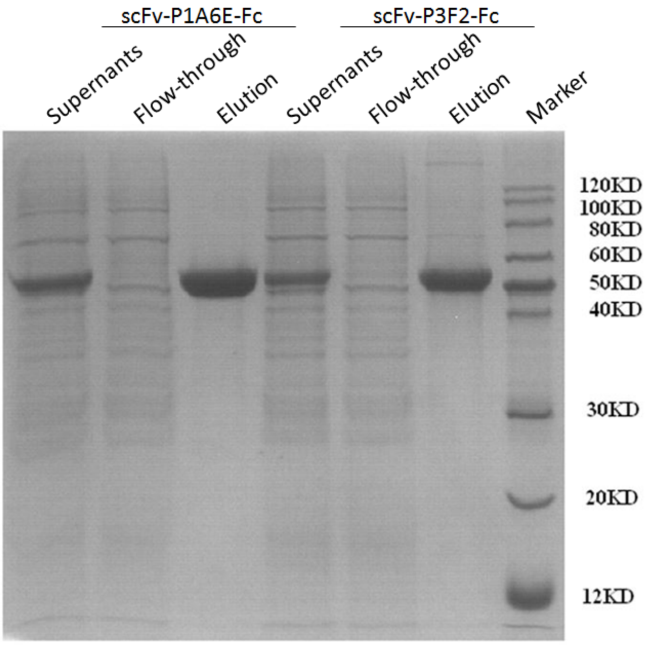


**Figure S1. Expression and purification of scFv-P1A6E-Fc and scFv-P3F2-Fc.** ScFv-Fc proteins were produced transiently in FreeStyle™ 293F cells. After the cells were cultured for 6-7 days, proteins were harvested by centrifugation and the cell culture supernatant was purified by protein A affinity chromatography. The purified protein were analyzed by SDS-PAGE.

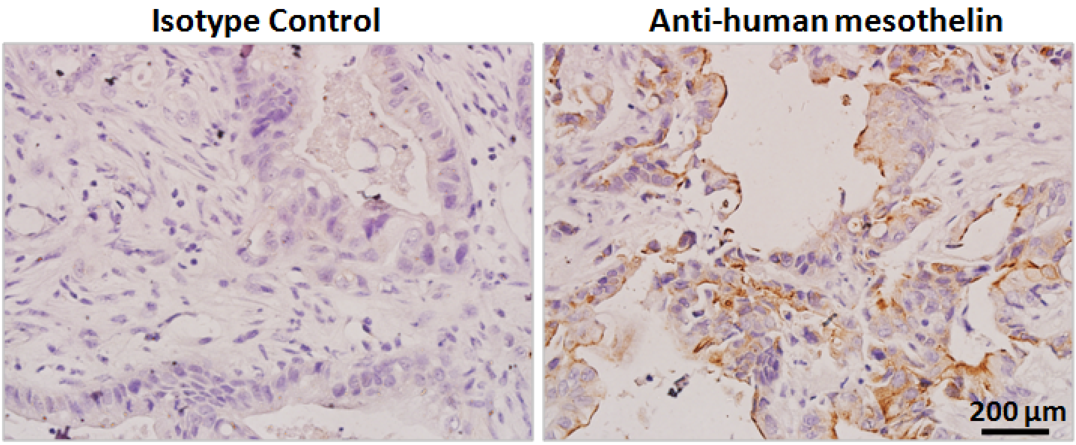


**Figure S2. The immunostaining of mesothelin on pancreatic cancer PDX tumors.** The mesothelin is over-expressed on the pancreatic cancer PDX tumors.

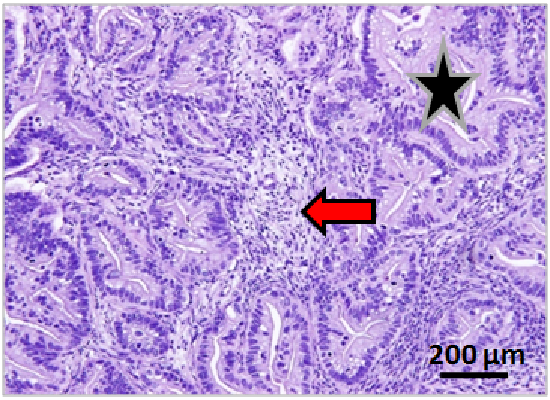


**Figure S3. The stroma of pancreatic cancer PDX tumors**. As shown in figure, the stroma percentage is about 30% in pancreatic cancer PDX tumors of passage six. Tumor ( ★ ) , stroma ( ⇦ )
